# Utilizing an Interprofessional Value Scale to Optimize Team Assessment

**DOI:** 10.1093/geroni/igaa057.014

**Published:** 2020-12-16

**Authors:** Jennifer Mendez, Atta Illahi, David Cicala, Martha Schiller, Shanique Brown

**Affiliations:** 1 Wayne State University, Detroit, Michigan, United States; 2 Wayne State University - School of Medicine, Detroit, Michigan, United States; 3 Wayne State University, Pharmacy & Health Science, Detroit, United States

## Abstract

Wayne State University Interprofessional Team Visit (IPTV) is a program that introduces medical, nursing, occupational therapy, pharmacy, physical therapy, physician assistant, dental, and social work students to working in a collaborative team during an older adult home visit assessment. This 60-minute visit, students ask questions about the older adult’s daily activities, nutrition, medications, family health, and social supports. Interprofessional education is crucial for health care delivery, there is a need to evaluate the engagement of students in teams. The Interprofessional Socialization and Valuing Scale (ISVS), measures self-perceived experiences with collaborative teamwork, including the ability, value, and comfort in working with others. The ISVS tool was used to evaluate educational experiences of students. There are 18 questions pertaining to perceptions of what students have learned from other disciplines. Students respond to each statement using a 7-point scale with 1 = “Not at All” and 7 = “To a Very Great Extent.” The Pre and Post data discuss the differences between groups of students in feeling comfortable working in teams. The feedback ensure better care, value, and education of future healthcare practitioners.

